# Fucoidan from *Undaria pinnatifida* Enhances Exercise Performance and Increases the Abundance of Beneficial Gut Bacteria in Mice

**DOI:** 10.3390/md22110485

**Published:** 2024-10-29

**Authors:** Cheng Yang, Corinna Dwan, Barbara C. Wimmer, Richard Wilson, Luke Johnson, Vanni Caruso

**Affiliations:** 1 School of Pharmacy and Pharmacology, University of Tasmania, Hobart, TAS 7005, Australia; cheng.yang@utas.edu.au; 2 Marinova Pty Ltd., 249 Kennedy Drive, Cambridge, TAS 7170, Australia; corinna.dwan@marinova.com.au (C.D.); barbara.wimmer@marinova.com.au (B.C.W.); 3 Central Science Laboratory, College of Science and Engineering, University of Tasmania, Hobart, TAS 7001, Australia; richard.wilson@utas.edu.au; 4 School of Psychological Sciences, Psychology, University of Tasmania, Launceston, TAS 7248, Australia; lukejohnsonphd@gmail.com

**Keywords:** fucoidans, *Undaria pinnatifida*, exercise performance, gut microbiome, Firmicutes/Bacteroidetes ratio, mitochondrial biogenesis, IGF1, COX4, MYH1

## Abstract

Fucoidans, known for their diverse biological properties such as anti-inflammatory, antiviral, antitumor, and immune stimulatory effects, have recently gained attention for their potential benefits in exercise endurance, muscle mass, and anti-fatigue. However, the mechanisms by which fucoidans enhance exercise performance are still unclear. To investigate these effects, we administered 400 mg/kg/day of fucoidan extract derived from *Undaria pinnatifida* to 64 C57BL/6J mice over 10 weeks. We evaluated changes in running activity, mitochondrial-related gene expression in skeletal muscle, and alterations in the intestinal microbiome. Our results showed that fucoidan supplementation significantly increased daily running distance and muscle mass by 25.5% and 10.4%, respectively, in mice on a standard chow diet, and with more modest effects observed in those on a high-fat diet (HFD). Additionally, fucoidan supplementation led to a significant increase in beneficial gut bacteria, including *Bacteroides/Prevotella*, *Akkermansia muciniphila*, and *Lactobacillus*, along with a notable reduction in the Firmicutes/Bacteroidetes ratio, indicating improved gut microbiome health. Mechanistically, fucoidan supplementation upregulated the mRNA expression of key genes related to mitochondrial biogenesis and oxidative capacity, such as COX4, MYH1, PGC-1α, PPAR-γ, and IGF1, in both standard chow and HFD-fed mice. Our findings suggest that fucoidan supplementation enhances exercise performance, improves muscle function, and positively modulates the gut microbiome in mice, regardless of diet. These effects may be attributed to fucoidans’ potential prebiotic role, promoting the abundance of beneficial gut bacteria and contributing to enhanced exercise performance, increased muscle strength, and improved recovery.

## 1. Introduction

Fucoidans are complex polysaccharides primarily extracted from various species of marine brown algae. They contain substantial amounts of L-fructose and sulphate ester groups [1,2]. Over the past decades, fucoidans have been extensively studied for their wide range of biological properties, including anti-inflammatory, antiviral, and antitumor as well as immune stimulatory activities [3,4,5,6,7,8].

In the field of exercise performance, recent interest has focused on the biological role of fucoidan, which demonstrated beneficial effects on exercise endurance, muscle mass and function, and anti-fatigue actions in mice [9,10,11]. Three weeks of *Laminaria japonica* fucoidan supplementation (310 and 620 mg/kg/day) increased grip strength and endurance swimming time in a dose-dependent manner, suggesting that fucoidan possesses a wide spectrum of bioactivities that can improve exercise performance and exhibit anti-fatigue effects [9]. In a later study, eight weeks of *Undaria pinnatifida* fucoidan (UPF) supplementation (0.25% of diet weight) enhanced mitochondrial biogenesis, increased oxidative muscle fibre, and promoted angiogenesis in skeletal muscles, resulting in increased treadmill distance and skeletal muscle mass [11]. Similarly, four weeks of oral administration of a UPF and a *Fucus vesiculosus* fucoidan (FVF) blend (400 mg/kg/day) increased muscle size and strength in both exercised and non-exercised mice, suggesting an important influence of fucoidan on skeletal muscle physiology [10].

Clinical trials in athletes consuming fucoidan demonstrated that the bioactive compound may influence various aspects of human physiology. For instance, in a double blind randomised controlled clinical trial, two weeks of UPF supplementation (1 g/day) had no effects on exercise performance, but it had a modest effect on inflammatory cytokines [12]. In another study, a blend of UPF and FVF increased the concentration of faecal lysozyme [6], a protein known for its antimicrobial and anti-inflammatory properties, suggesting that fucoidan plays a potential role in protecting mucosal barrier integrity [13].

Additionally, in recent years, researchers have produced a significant amount of literature dedicated to understanding how fucoidans influence the gut microbiome’s composition and function. Several studies suggest that fucoidan induces favourable microbiota alterations by exerting prebiotic effects [2,14], and the scientific evidence suggests a link between fucoidan, diet, exercise performance and composition of the gut microbiome [6,15,16,17]. In mice fed a high fat diet (HFD), 8 weeks of UPF supplementation alleviated dyslipidaemia, decreasing the total serum cholesterol, LDL cholesterol (LDL-C), and liver cholesterol levels as well as modulating the gut microbiota [18]. Similarly, 5 weeks of fucoidan administration reduced obesity and improved gut microbiota in mice [19].

Regular exercise can enrich the microflora diversity improving the development of beneficial bacteria, including *Bifidobacterium* and *Akkermansia*, which are linked to gut health for their anti-inflammatory properties and their positive impact on gut barrier integrity [20,21]. Exercise can also modify the ratio of two dominant phyla, *Firmicutes* and *Bacteroidetes*, which has been associated with several diseases, including diabetes, gut and brain health, and some cancers [22,23]. In obese mice, a 12-week free running wheel exercise intervention ameliorated the metabolic consequences caused by HFD consumption and changed the gut microbiome composition, increasing the abundance of *Bacteroides* while decreasing *Firmicutes* [24].

Based on the effects of UPF on the gut microbiome and skeletal muscle, in the current study, we aimed to examine whether UPF could mitigate the negative impact of HFD consumption on exercise performance while increasing the abundance of beneficial gut bacteria. For this purpose, UPF was administered orally to both male and female mice for 10 weeks. The daily voluntary running activity and changes in intestinal microbiome were assessed. To further investigate the mechanisms involved, the expression of genes involved in mitochondrial activity and energy utilization in skeletal muscle that influence contraction speed, endurance, and metabolic adaptation to exercise were measured [11,25]. Specifically, markers such as mitochondrial cytochrome c oxidase subunit 2 and 4 (COX2 and COX4) as well as muscle myosin heavy chain 1 (MYH1) were measured.

## 2. Results

### 2.1. Effects of UPF on Running Activity and Muscle Mass

**Figure 1:**
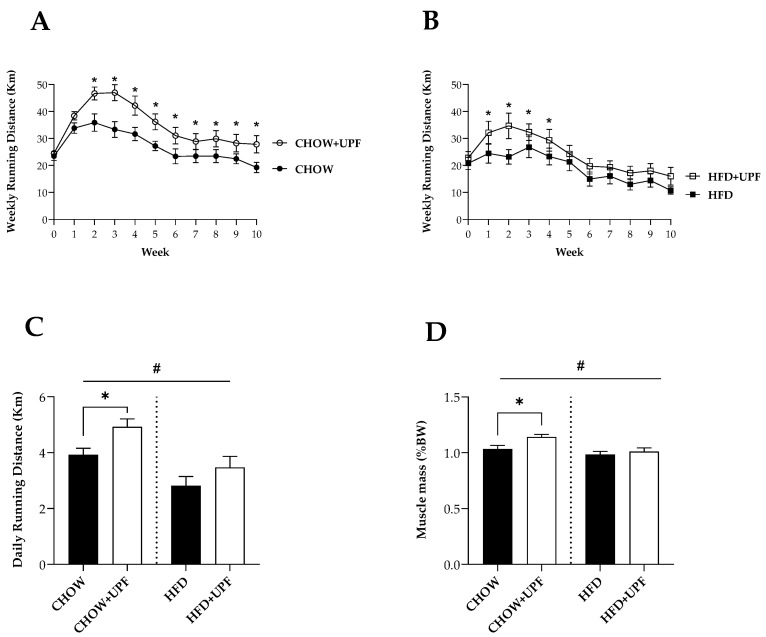
Effects of UPF on running activity and muscle mass. (**A**) Changes in running activity of mice consuming a standard chow diet (CHOW) (black round, *n* = 10), and those treated with UPF (open round, *n* = 10). (**B**) Changes in running activity of mice consuming a HFD (black square, *n* = 10), and those treated with UPF (open square, *n* = 10). (**C**) Daily running activity (*n* = 10). (**D**) Muscle mass of mice at sacrifice calculated as percentage of wet weight to body weight (*n* = 15–16). Results are expressed as mean ± SEM. In graphs (**A**,**B**), data were analysed by repeated measures two-way ANOVA with UPF treatment and weeks of treatment as factors. In graphs (**C**,**D**), data were analysed by one-way ANOVA followed by a post hoc assessment using Fisher’s least significant difference test (LSD). #, significant difference for overall diet effect CHOW and CHOW + UPF vs. HFD and HFD + UPF (*p* < 0.05); *, significant difference for UPF effect (*p* < 0.05).

In mice consuming a standard chow diet, weekly running distance differences reached statistical significance after two weeks of UPF treatment and was maintained until the end of the experiment (*p* < 0.05, Figure 1A).

In mice consuming a HFD, weekly running distance differences reached statistical significance after 1 week of UPF treatment and was maintained for the following 4 weeks (*p* < 0.05, Figure 1B).

Mice consuming a chow diet ran significantly greater distances daily than those consuming a HFD (Figure 1C). Specifically, in mice consuming a standard chow diet, UPF significantly increased daily running activity compared to the control group (25.5%; *p* < 0.05; Figure 1C). UPF did not improve running activity in mice consuming a HFD.

Mice consuming a chow diet together with UPF showed significantly higher muscle mass than mice consuming a chow diet only (10.4%; *p* < 0.05; Figure 1D). This difference was not observed for mice on a HFD with or without fucoidan consumption.

Data on the effects of UPF, diet and exercise on energy intake and anthropometric parameters including blood glucose, body fat, muscle, plasma leptin and plasma ghrelin are shown in Supplementary Table S5.

### 2.2. Effects of UPF on Total DNA Content in the Faeces

**Figure 2:**
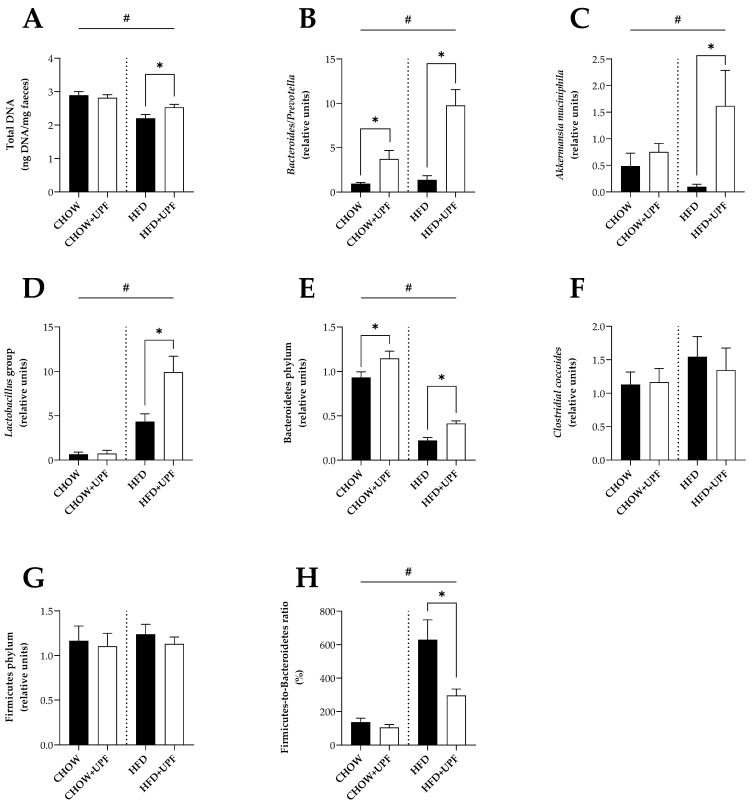
Total DNA content and abundance of representative bacterial groups and species in mice faeces. Total DNA content is expressed as ng DNA per mg of faeces. (**A**) Total DNA content in the faeces (*n* = 15–16) after 10 weeks of UPF administration. DNA abundance of 16S rRNA gene of representative bacterial groups/species (**B**) *Bacteroides/Prevotella*, (**C**) *Akkermansia muciniphila*, (**D**) *Lactobacillus* group, (**E**) Bacteroidetes phylum, (**F**) *Clostridial coccoides*, and (**G**) Firmicutes phylum were analysed in mouse faeces and normalised with the average of the CHOW group (2^−ΔΔCt^). Bacterial quantities are expressed as relative units. And (**H**) the ratio of Firmicutes to Bacteroidetes was calculated by dividing the relative DNA expression value of Firmicutes by the relative DNA expression value of Bacteroidetes. *n* = 7–10. Results are presented as mean ± SEM. In the graphs (**A**–**H),** data were analysed by one-way ANOVA, followed by post hoc LSD tests. #, *p* < 0.05, overall difference between CHOW and CHOW + UPF vs. HFD and HFD + UPF. *, *p* < 0.05, difference between CHOW vs. CHOW + UPF and HFD vs. HFD + UPF.

Overall, mice consuming a chow diet had higher DNA content in the faeces (20.6%) compared to mice consuming a HFD (Figure 2A). In mice consuming a HFD, UPF increased the total DNA content (14.8%), (Figure 2A, *p* < 0.05), while the compound had no effect on mice consuming a chow diet.

#### Effects of Diet and UPF Administration on Gut Microbial Profiling

Overall, *Bacteroides/Prevotella*, *Akkermansia muciniphila*, and the *Lactobacillus* group (Figure 2B–D) had higher DNA abundance in mice fed a HFD. Conversely, in the phylum Bacteroidetes (Figure 2E), the HFD group had significantly lower DNA abundance compared to the CHOW groups.

In mice eating a standard chow diet, UPF supplementation significantly increased the DNA levels of *Bacteroides*/*Prevotella* (*p* < 0.05, Figure 2B) and the phylum Bacteroidetes (*p* < 0.05, Figure 2E).

In mice consuming a HFD, animals receiving UPF showed a significant increase in two bacterial species, including *Bacteroides/Prevotella* (*p* < 0.05, Figure 2B) and *Akkermansia muciniphila* (*p* < 0.05, Figure 2C). Likewise, the administration of UPF induced a significant increase in the *Lactobacillus* group (*p* < 0.05, Figure 2D) and Bacteroidetes phylum (*p* < 0.05, Figure 2E). However, consuming UPF was not associated with a change in the abundance of bacterial *Clostridial coccoides* (Figure 2F) and Firmicutes (Figure 2G).

In contrast, in mice receiving UPF, the ratio of Firmicutes to Bacteroidetes phylum in the faeces of mice consuming a HFD was significantly reduced (*p* < 0.05, Figure 2H), but no UPF effect was observed in the chow group.

### 2.3. Correlation of Gut Microbial Abundance with Mouse Body Weight, Visceral Fat, and Running Activity

When the abundance of specific bacterial groups/species in mouse faecal contents were plotted against body weight, visceral fat (gonadal and retroperitoneal fat pads), and daily running distance, Bacteroidetes phylum (*r* = −0.708; *p* = 0.0001; *n* = 10) and *Akkermansia muciniphila* (*r* = −0.399; *p* = 0.0354; *n* = 7) were found to be significantly negatively correlated with body weight. In addition, visceral fat was negatively correlated with the phylum Bacteroidetes (*r* = −0.779; *p* = 0.0001; *n* = 10). Conversely, running distance showed significantly positive correlations with the Bacteroidetes phylum (*r* = 0.598; *p* = 0.0001; *n* = 10) and *Akkermansia muciniphila* (*r* = 0.420; *p* = 0.0259; *n* = 7); however, a negative correlation with running distance was found for *Clostridial coccoides* (*r* = −0.430; *p* = 0.0089; *n* = 9). From the perspective of the F/B (Firmicutes to Bacteroidetes) ratio, body weight (*r* = 0.568; *p* = 0.0001; *n* = 10) and visceral fat (*r* = 0.593; *p* = 0.0001; *n* = 10) showed significantly positive correlations; conversely, the running distance was negatively correlated with the F/B ratio (*r* = −0.559; *p* = 0.0002; *n* = 10). The correlation matrix is presented in Table 1.

### 2.4. Effects of Diet and UPF on Muscle Gene Expression

**Figure 3:**
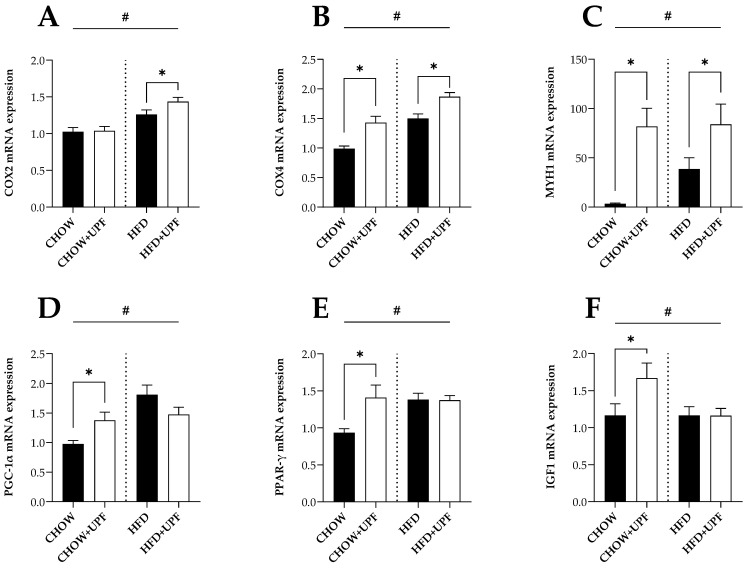
Effects of UPF on muscle gene expression. mRNA levels of (**A**) COX2, (**B**) COX4, (**C**) MYH1, (**D**) PGC-1α, (**E**) PPAR-γ, and (**F**) IGF1. *n* = 13–16. Results are expressed as mean ± SEM. Data were analysed by one-way ANOVA, followed by post hoc LSD tests. #, *p* < 0.05, overall difference between CHOW and CHOW + UPF vs. HFD and HFD + UPF. *, *p* < 0.05, difference between CHOW vs. CHOW + UPF and HFD vs. HFD + UPF.

Overall, HFD increased the muscle mRNA expression of cytochrome c oxidase subunit 2 and 4 (COX2 and COX4, Figure 3A,B), myosin heavy chain 1 (MYH1, Figure 3C), peroxisome proliferator-activated receptor gamma coactivator 1α (PGC-1α, Figure 3D), peroxisome proliferator-activated receptor gamma (PPAR-γ, Figure 3E), and insulin-like growth factor 1 (IGF1, Figure 3F) compared with a standard chow diet.

In mice eating a standard chow diet, the administration of UPF induced a significant increase in the mRNA expression of COX4 (*p* < 0.05, Figure 3B), MYH1 (*p* < 0.05, Figure 3C), PGC-1α (*p* < 0.05, Figure 3D), PPAR-γ (*p* < 0.05, Figure 3E), and IGF1 (*p* < 0.05, Figure 3F).

In mice consuming a HFD, UPF supplementation significantly increased the mRNA expression of COX2 (*p* < 0.05, Figure 3A), COX4 (*p* < 0.05, Figure 3B), and MYH1 (*p* < 0.05, Figure 3C).

## 3. Discussion

The current study identified potential mechanisms by which UPF may enhance exercise performance, focusing on changes in the abundance of beneficial gut bacteria and alterations in skeletal muscle gene expression. Over the 10-week experimental period, UPF supplementation (400 mg/kg/day) increased voluntary running activity in mice on both a standard chow diet and a high-fat diet (HFD). In mice on the standard chow diet, two weeks of UPF treatment enhanced running distance, with this effect sustained throughout the experiment. Additionally, mice that ingested UPF had an increase in muscle mass of 10.4% compared to the untreated group. In the HFD group, mice that ingested UPF also had a significantly increased running distance, but this effect lasted only five weeks and there was no impact on muscle mass. The results also showed that UPF upregulated the key genes involved in mitochondrial function and energy homeostasis, including COX4, MYH1, PGC-1α, PPAR-γ, and IGF1, and these effects were associated with changes in the abundance of some beneficial gut bacteria belonging to the Firmicutes and Bacteroides phyla. In our study, 10 weeks of fucoidan supplementation had no effect on food intake, body weight, or adipose tissue mass, including gonadal, visceral, retroperitoneal, and brown adipose tissue (Supplementary Table S5). These parameters were consistent with our biochemical assessments, which showed no changes in glucose, leptin, or ghrelin levels throughout the experiment (Supplementary Table S5). Fucoidans are known primarily for their anti-inflammatory, antioxidant, and immune-modulating properties rather than their direct effects on energy balance or fat metabolism [8,26]. These characteristics may explain why UPF did not influence key metabolic markers like glucose, leptin, or ghrelin, which regulate appetite, energy storage, and hunger signals.

To build upon previous studies investigating the exercise performance-enhancing properties of UFP [6,9,10], we aimed to elucidate its potential mechanism of action. Our results suggest that UPF may improve exercise performance by modifying the abundance of beneficial gut bacteria, which are known to influence muscle strength and energy utilization [17,27,28].

According to several studies, fucoidans promote beneficial effects on exercise endurance, muscle mass and function, and anti-fatigue actions [9,10,11]. Consistent with these findings, we confirmed that UPF increased weekly running activities in mice on both chow and high-fat diets, with a more pronounced effect in those on a chow diet. This effect emerged after just two weeks of treatment and persisted throughout the experiment, whereas in the high-fat diet group, the effect lasted four weeks. We also noted that the increase in running activity was accompanied by an increase in muscle mass, but this was observed only in mice consuming a chow diet. Our results are in line with previous results, where three weeks of fucoidan supplementation derived from *Laminaria japonica* (620 mg/kg/day) improved the exercise performance of male mice in a dose-dependent manner [9]. The results of the study showed that fucoidan significantly increased grip strength and endurance swimming time and that this treatment led to dose-dependent reductions in exercise-induced fatigue-related parameters including serum lactate and ammonia levels, along with an increase in glucose levels following a 15 min swimming test [9]. Similarly, four weeks of UPF and *Fucus vesiculosus* fucoidan (400 mg/kg/day) increased muscle size and strength in both exercised and non-exercised mice, suggesting an important influence of fucoidan on skeletal muscle physiology [10]. 

In humans, several physiological effects of fucoidan consumption have been observed in athletes. For example, in a clinical trial, two weeks of fucoidan supplementation (1  g/day) had no effects on exercise performance, but it promoted a modest effect on inflammatory cytokines [12]. In another study, fucoidan increased the concentration of faecal lysozyme [6], a protein known for its antimicrobial and anti-inflammatory properties, suggesting that fucoidan plays a potential role in protecting mucosal barrier integrity [13]. Collectively, our findings and previous research suggest that long-term fucoidan supplementation may offer broad health benefits, including performance enhancement and anti-fatigue effects.

On the basis of these results, we tested the effects of UPF on key genes involved in mitochondrial function and energy homeostasis, including COX4, MYH1, PGC-1α, PPAR-γ, and IGF1 in skeletal muscle. In mice consuming a standard chow diet or a HFD, UPF significantly upregulated the mRNA expression of COX4, MYH1, PGC-1α, PPAR-γ, and IGF1, indicating improved mitochondrial biogenesis and oxidative capacity [29,30].

COX2 and COX4 are integral subunits of respiratory chain complex IV in the mitochondrial electron transport chain; COX2, encoded by mitochondrial DNA, forms the catalytic core essential for electron transfer and oxygen reduction; while COX4, encoded by nuclear DNA, modulates the enzyme’s activity and efficiency [31]. The synergism between COX2 and COX4 ensures effective oxidative phosphorylation (OXPHOS) and ATP synthesis, which are vital for cellular energy production [32]. The upregulation of COX2 and COX4 in this study supported the hypothesis that UPF enhances mitochondrial function and oxidative metabolism [25], crucial for maintaining muscle efficiency under metabolic stress [32,33]. Additionally, we report that UPF increases MYH1 expression, which is central for maintaining muscle fibre composition and structural integrity during endurance activities in physiological conditions [34,35]. Furthermore, increased PPAR-γ expression suggests a potential role in regulating lipid metabolism within muscle tissues, which may contribute to improved muscle performance and adaptation [36]. These effects likely explain the observed improvements in voluntary running activity and muscle mass in the UPF-treated chow group, suggesting that UPF supports enhanced muscle function and endurance [11,37]. Collectively, our findings suggest that UPF may enhance muscle energy metabolism and improve fatigue resistance by modulating genes involved in mitochondrial biogenesis, oxidative metabolism, and muscle growth across both standard chow and high-fat diet contexts. Further research is needed to elucidate the molecular mechanisms and assess the long-term benefits of UPF supplementation on muscle function and metabolic health.

In addition to previous studies investigating the impacts of UPF on exercise performance and muscle function [9,10], the present study aimed to elucidate how 10 weeks of UPF supplementation would influence the changes in the gut microbiome, and specifically on the abundance of Firmicutes and Bacteroides phyla.

It is interesting to note that in our study, mice consuming a HFD supplemented with UPF exhibited a 14.9% increase in total DNA content compared to their control group. The change in DNA content that we observed could be explained, at the least in part, by the evidence that exercise training alters the composition and functional capacity of the gut microbiota independently of the diet consumed [15,16,22,38,39,40]. Additionally, other studies demonstrated that the magnitude of the changes in bacterial DNA may be related to the obesity status as well as exercise intensity and modality [17,28,39,41].

In our study, we investigated the abundance of *Akkermansia muciniphila*, a bacterium widely accepted as a biomarker of intestinal health because of its numerous beneficial properties, such as improved glucose metabolism, enhanced insulin sensitivity, and reduced inflammation [42,43,44]. Here, we report that while a chow diet did not affect the abundance of *Akkermansia muciniphila*, mice on a high-fat diet showed reduced levels of this bacterium. However, UPF supplementation significantly increased *Akkermansia muciniphila* abundance, suggesting a prebiotic role in promoting the growth of beneficial gut bacteria [14,45,46].

We also measured the abundance of the *Lactobacillus* group, a bacterium that plays a major role in human health by reducing the release of cortisol, decreasing inflammatory cytokines, and improving the immune system [47,48]. The *Lactobacillus* group is considered to a probiotic agent itself because it helps to create a favourable gut environment [47], lowering the gut pH and inhibiting the growth of harmful intestinal bacteria [47,48]. In mice fed a standard chow diet and treated with UPF, the levels of *Lactobacillus* remained unchanged. However, in those consuming a HFD, the abundance of *Lactobacillus* increased, and the treatment with UPF further augmented the Lactobacillus levels within this group. Additionally, UPF increased the abundance of *Bacteroides*/*Prevotella*, bacterial species recognized for their prebiotic effects [49], suggesting and reinforcing a potential prebiotic role of UPF.

A novel finding of our study is that UPF positively altered the Firmicutes-to-Bacteroidetes (F/B) ratio. Firmicutes and Bacteroidetes represent the predominant gastrointestinal bacterial phyla, collectively constituting over 90% of the gut microbiota [50]. The F/B ratio has been linked to the maintenance of healthy intestinal homeostasis, and disruption of this balance can result in several serious complications in both humans and animals [51,52,53]. For instance, obese individuals typically exhibit an elevated F/B ratio compared to healthy counterparts [41,54]. Conversely, patients with breast cancer tend to have an F/B ratio three times lower than that of healthy controls [53]. Inflammatory bowel disease is also associated with a low F/B ratio, while benign prostatic hyperplasia is linked to a high F/B ratio [51,52].

In our study, and in line with previous research in which HFD consumption increased the phylum Firmicutes and decreased Bacteroidetes [55], mice consuming a HFD had a significant higher F/B ratio. However, 10 weeks of oral UPF supplementation significantly lowered this ratio compared to the control group, suggesting a potential benefit of the supplement in modulating beneficial gut bacteria. We also found that the F/B ratio was positively correlated with body weight, and negatively with running activity as demonstrated in previous studies where exercise reduced the F/B ratio in obese mice [40]. Overall, our findings substantiated the effects of UPF on beneficial gut bacteria including *Akkermansia muciniphila* and *Lactobacillus* and on the F/B ratio, suggesting a prebiotic role of the supplement, especially in concomitance of HFD consumption.

While animal microbiota studies offer useful preliminary insights, translating these findings to human physiology has limitations due to differences in environment, species-specific diets, metabolic processes, and immune system variations [56]. Thus, to confirm the results observed in our study, it is essential to conduct clinical trials in the future.

Our targeted approach to analyse specific genes of interest has shed light on the potential impacts on mitochondrial and fibre function in muscle; however, global “omics” approaches such as proteomics and mRNA sequencing would lead to more comprehensive insight into the activity of UPF. Specifically, 16S rRNA gene sequencing would provide a more comprehensive analysis of microbial diversity and composition [56]. For future studies, this method would be able to identify not only the specific bacterial populations present but also quantify their relative abundances allowing for a deeper understanding of the gut microbiota’s interactions.

Furthermore, detailed characterization of the UPF constituents would facilitate investigation into the molecular mechanisms underlying any therapeutic effects of the supplement. It is widely recognized that the source of fucoidan is an important factor when comparing outcomes, as its properties can vary significantly depending on species, molecular weight fractions, and component percentages [57].

In our study, we found no significant sex differences in exercise performance or in the abundance of beneficial gut bacteria linked to enhanced exercise outcomes. Although several studies in mice have demonstrated that sex can influence microbial composition [58,59], findings on sex-related differences in gut microbiota remain inconsistent. Some research suggests that male mice may exhibit lower microbial diversity than females, while others indicate that factors such as species and mouse strain may have a more substantial impact than sex [60]. For instance, a study involving different mouse strains found that while sex contributed to microbiota variance, species and strain had a more pronounced effect [61]. It is well established that various factors, including diet, genetic background, age, environmental influences, and exercise, shape the gut microbiota [56]. While sex is one of the key variables affecting gut microbiota, it can also be a confounding factor. Therefore, further research is needed to clarify the relationship between sex differences and changes in the gut microbiome, particularly in the context of exercise performance.

In conclusion, based on the data presented in this study, we propose a descriptive mechanism by which 10 weeks of UPF supplementation enhanced exercise performance, upregulated muscle function-related genes, and modulated the gut microbiome abundance of beneficial bacteria in mice consuming a standard chow diet as well as those on a HFD. Our findings suggest that UPF may play a prebiotic role in the gut, contributing to an increase in the abundance of beneficial gut bacteria, which may result in enhanced exercise performance, increased muscle strength, or improved recovery times. Further research is needed to elucidate the underlying molecular mechanisms by which UPF exerts its beneficial therapeutic effects.

## 4. Materials and Methods

### 4.1. Ethics Statement

This investigation was authorised by the Animal Ethics Committee of the University of Tasmania (A0027164). The animal work and procedures in this study were executed in strict adherence to the provisions delineated in the Tasmanian Animal Welfare Act (1993/63) and the Australian Code of Practice for the Care and Use of Animals for Scientific Purposes 8th edition 2013 [62].

### 4.2. Animals and Diet

Four-week-old male and female C57BL/6J mice (*n* = 32 males and *n* = 32 females, Animal Services, University of Tasmania) were housed under controlled conditions at a temperature of 20 ± 2 °C and maintained on a standard 12:12 h light/dark cycle. Following a one-week acclimatization period, 32 mice were provided with *ad libitum* access to a commercial high-fat pelleted diet (HFD) (19.4 MJ/kg, 23.5% fat, 23% protein, 5.4% crude fibre, SF16-059, Specialty Feeds, Perth, Australia), while the control group (32 mice) received a standard chow diet (12.8 MJ/kg, 6% fat, 20% protein, 3.2% crude fibre, product code 102108, Ridly Agri-Products). Mice were singularly housed with unrestricted access to food and drinking water *ad libitum*. Each cage was equipped with a free-running wheel, shown in Supplementary Information (Figures S1 and S2), to assess the mice’s exercise performance and mitigate social stress associated with isolation. Throughout the study, running distance was recorded daily, while body weight and food intake were recorded weekly. Mice were euthanized after 10 weeks of UPF administration.

### 4.3. Experimental Groups

The 64 mice were randomly divided into four groups (*n* = 16): CHOW, CHOW + UPF, HFD, and HFD + UPF.

### 4.4. UPF Jelly Preparation

A fucoidan extract (≥85% fucoidan) derived from *Undaria pinnatifida* (UPF, Batch No.: UPF2022532) was provided by Marinova Pty Ltd. (Tasmania, Australia) and stored at ambient temperature (21–29 °C). The chemical composition of fucoidan used in this study is described in the Supplementary Information (Tables S1 and S2). The fucoidan was incorporated into an artificially flavoured and sweetened jelly for voluntary oral administration to mice by using 6% *w*/*v* gelatine (Ward McKenzie, Altona Australia) as the gelling agent, 9% *v*/*v* strawberry essence (Roberts Edible Craft, Keysborough, Australia) for flavour, and 2% *w*/*v* sucralose (Cat# 69293, Sigma–Aldrich, Bayswater, Australia) as a non-caloric sweetener. The test substance application in jelly has been used successfully in the past [6,8,63,64,65,66].

In the CHOW + UPF and HFD + UPF groups, each serving of jelly was prepared with a total volume of 200 µL, incorporating 12 mg of UPF (equivalent to 400 mg/kg/day). In the CHOW and HFD groups, each serving of jelly was prepared in the same manner but without the addition of fucoidan. Both UPF and vehicle jellies were served daily in small dishes. A dose of 400 mg/kg day was selected for this study based on previous fucoidan studies in mice [10,67] and consideration of the human equivalent dose (HED). Fucoidan has been shown to be safe and effective in a wide range of human clinical studies administered at oral doses of 1 g, 2.2 g and 3 g [6,68,69]. To calculate the HED, the following formula from the US Food and Drug Administration was used: assuming a human weight of 60 kg, the HED for 2 (g) ÷ 60 (kg) = 0.033 × 12.3 = a mouse dose of 400 mg/kg [70].

### 4.5. Training and Jelly Administration

Mice were trained to consume the jelly using a previously reported method [65] to ensure the mice were accustomed to its taste and texture. The jelly was prepared weekly and stored at 4 °C to maintain freshness. The jelly was consistently served to the mice at 9:00 am each day, alongside their regular food (standard chow diet or HFD) to ensure continuous food intake and prevent potential nutritional deficiencies. All animals consumed the full 200 µL jelly provided daily (Supplementary Video S1).

### 4.6. Sample Collection

After 10 weeks of drug administration, mouse faecal samples were collected in the morning and stored in a −80 °C freezer for the gut microbial profiling study. Mice were then fasted overnight (12 h) and euthanized via carbon dioxide inhalation. Blood was collected by cardiac puncture and centrifuged (12,000× *g* rpm/10 min). Plasma was separated and stored at −80 °C for analysis of the metabolic markers. Visceral fat pads (gonadal and retroperitoneal fat) and skeletal muscle (gastrocnemius) were dissected, weighed, snap-frozen in liquid nitrogen, and stored in a −80 °C freezer until further analysis.

### 4.7. Gut Microbial Profile

Total bacterial DNA was extracted from approximately 100 mg of mice faecal samples after 10 weeks of UPF administration using QIAamp PowerFecal Pro DNA Kits (Cat# 51804, Qiagen, Chuo-ku, Japan) according to the manufacturer’s instructions and stored at −80 °C until further processing. DNA concentration and purity ratios (A260/280 and A260/230) were measured by a Nanodrop™ 8000 Spectrophotometer (NanoDrop Technologies Inc., Minato-ku, Japan) and only samples with an absorbance ratio of ~1.8 were used for quantitative polymerase chain reaction (qPCR) assays.

Specific primers for Firmicutes phylum, Bacteroidetes phylum, Enterobacteriales order, *Bifidobacterium* spp., *Lactobacillus* group, *Bacteroides/Prevotella*, *Akkermansia muciniphila*, *Clostridial coccoides*, and Total Bacteria were obtained from Sigma–Aldrich (Supplementary Table S3). The abundance of specific bacterial groups and species was determined by measuring DNA expression of the 16S rRNA gene sequences by qPCR with the CFX Connect ^TM^ Real-Time PCR Detection system (Bio-Rad, South Granville, Australia), following previously described protocols [55,71].

The reactions were conducted with 2.5 μL DNA (2 ng/μL), 1.5 μL nucleus-free distilled water, 0.5 μL forward primer (10 μM), 0.5 μL reverse primer (10 μM), and 5 μL PowerUp SYBR Green Master Mix (Cat# A25918, Applied Biosystems) in a total volume of 10 μL. A total of 2.5 μL of nucleus-free distilled water (Cat# 10977015, Thermo Fisher Scientific, Scoresby, Australia) was used as a negative control in each run. Target bacterial DNA expression was normalized against Total Bacteria (housekeeping gene) using the average of the control group as a calibrator, as previously described [71]. Analysis was performed using the 2^−ΔΔCt^ method [72].

### 4.8. Real-Time Quantitative PCR (RT-qPCR) Assay

Frozen skeletal muscle tissue was used to extract and purify total RNA using an RNeasy Mini Kit (Cat# 74106, Qiagen, Chuo-ku, Japan) according to the manufacturer’s recommendations. A Nanodrop^TM^ 8000 Spectrophotometer (NanoDrop Technologies Inc., Minato-ku, Japan) was used to measure the RNA concentration and purity ratios (A260/280 and A260/230) and only samples with an absorbance ratio of ~2.0 were used for complementary DNA (cDNA) synthesis. One microgram of RNA template from each sample was reverse transcribed into cDNA using a High-Capacity cDNA Reverse Transcription Kit (Cat# 4368814, Applied Biosystem, Waltham, MA, USA). All primers were purchased from Kicqstart SYBR Green Primers (Sigma-Aldrich, St. Louis, MO, USA). The sequences of the primers used for quantification of gene expression of COX2, COX4, MYH1, IGF1, PGC-1α, PPAR-γ, β-actin, and GAPDH can be found in Supplementary Table S4.

RT-qPCR reactions were performed using PowerUp SYBR Green Master Mix (Cat# A25918, Applied Biosystems, Waltham, MA, USA) in the CFX Connect TM Real-Time PCR Detection system (Bio-Rad) following the manufacturer’s instructions. The amplification program included an initial denaturation step at 95 °C for 5 min, followed by 40 cycles of 10 s at 95 °C, 10 s at 60 °C, and 20 s at 72 °C. After each amplification, a melting curve study was conducted to validate the reliability of the results and product specificity. Target gene expression was normalized against β-actin and GAPDH housekeeping genes using the average of control group as a calibrator. Analysis was performed using the comparative 2^−ΔΔCt^ method [72].

### 4.9. Statistical Analysis

Statistical analysis was performed using GraphPad Prism version 8.3.0 for Windows (GraphPad Software, San Diego, CA, USA, www.graphpad.com, accessed on 31 August 2024) and the results were expressed as mean ± SEM. Body weight change and weekly running distance were analysed by two-way repeated measures ANOVA with a fucoidan effect and time points as factors. The effects of fucoidan on the final body weight, energy daily intake, visceral adipose fat, skeletal muscle, and gut microbial DNA expression levels were performed by one-way ANOVA. Data were then assessed by a post hoc analysis using Fisher’s least significant difference test (LSD) as appropriate. The results were considered statistically significant when *p* < 0.05.

## Figures and Tables

**Table 1:** Correlations between the abundance of bacterial groups/species in faeces with mouse body weight, visceral fat, and running distance.

Bacterial Groups/Species	Body Weight		Visceral Fat		Running Distance	
	*r*	*p*	*r*	*p*	*r*	*p*
Bacteroidetes phylum	0.708 **	0.0001	0.779 **	0.0001	0.598 **	0.0001
Firmicutes phylum	0.136	0.4022	0.111	0.4950	0.261	0.1035
*Clostridial coccoides*	0.107	0.5362	0.217	0.2039	0.430 **	0.0089
*Akkermansia muciniphila*	0.399 *	0.0354	0.140	0.4756	0.420 *	0.0259
F/B ratio	0.568 **	0.0001	0.593 **	0.0001	0.559 **	0.0002

Linear relationships were tested using Pearson’s correlation coefficients (*r*). Significant correlations are marked as follows: *, *p* < 0.05; **, *p* < 0.01. F/B ratio: Firmicutes to Bacteroidetes ratio. Visceral fat: gonadal and retroperitoneal fat pads.

## Data Availability

The raw data supporting the conclusions of this article will be made available by the authors on request.

## Supplementary Materials

The following supporting information can be downloaded at: https://www.mdpi.com/article/10.3390/md22110485/s1, Figure S1: Running Wheel; Figure S2: Running Distance analysis; Table S1: Absolute mass percentages of *Undaria pinnatifida* fucoidan (UPF) extract; Video S1: Effective Voluntary Oral Drug Administration in Mice: Drug-Containing/Vehicle Jelly; Table S2: Carbohydrate breakdown (mass %) of neutral carbohydrates in *Undaria pinnatifida* fucoidan (UPF) extract; Table S3: Sequence of primers used for RT-qPCR assay in mouse muscle samples; Table S4: Sequence of primers used for bacterial profiling by quantitative PCR; Table S5: Effects of UPF, diet and exercise on energy intake and anthropometric parameters.
