# Supplementary material for: Fucoidan from Undaria pinnatifida Enhances Exercise Performance and Increases the Abundance of Beneficial Gut Bacteria in Mice
Source: Mar Drugs. 2024 Oct 29;22(11):485. doi: 10.3390/md22110485 (PMC11595500; doi:10.3390/md22110485)
Supplement: Supplementary file 1 [file marinedrugs-22-00485-s001.zip › marinedrugs-3238273-supplementary.pdf]

Supplementary Materials

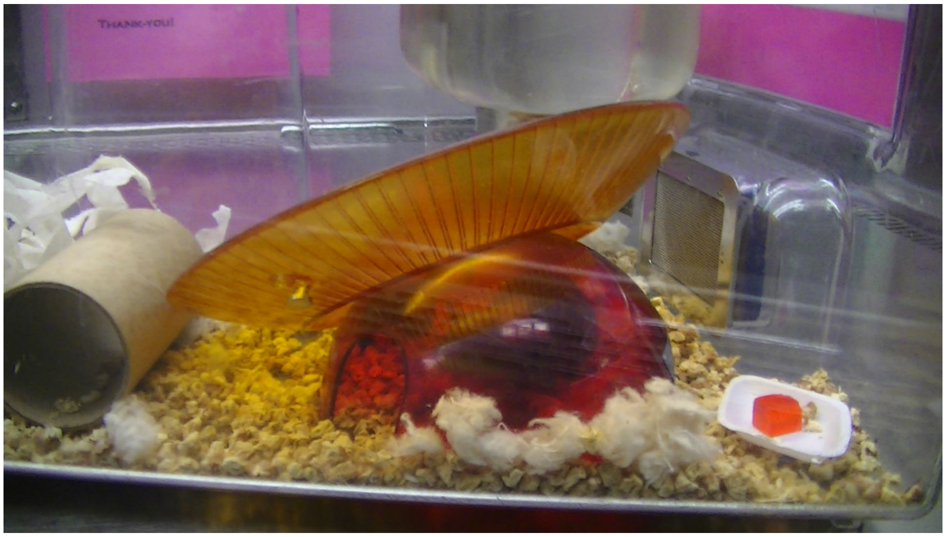

Figure S1. Sample of running Wheel

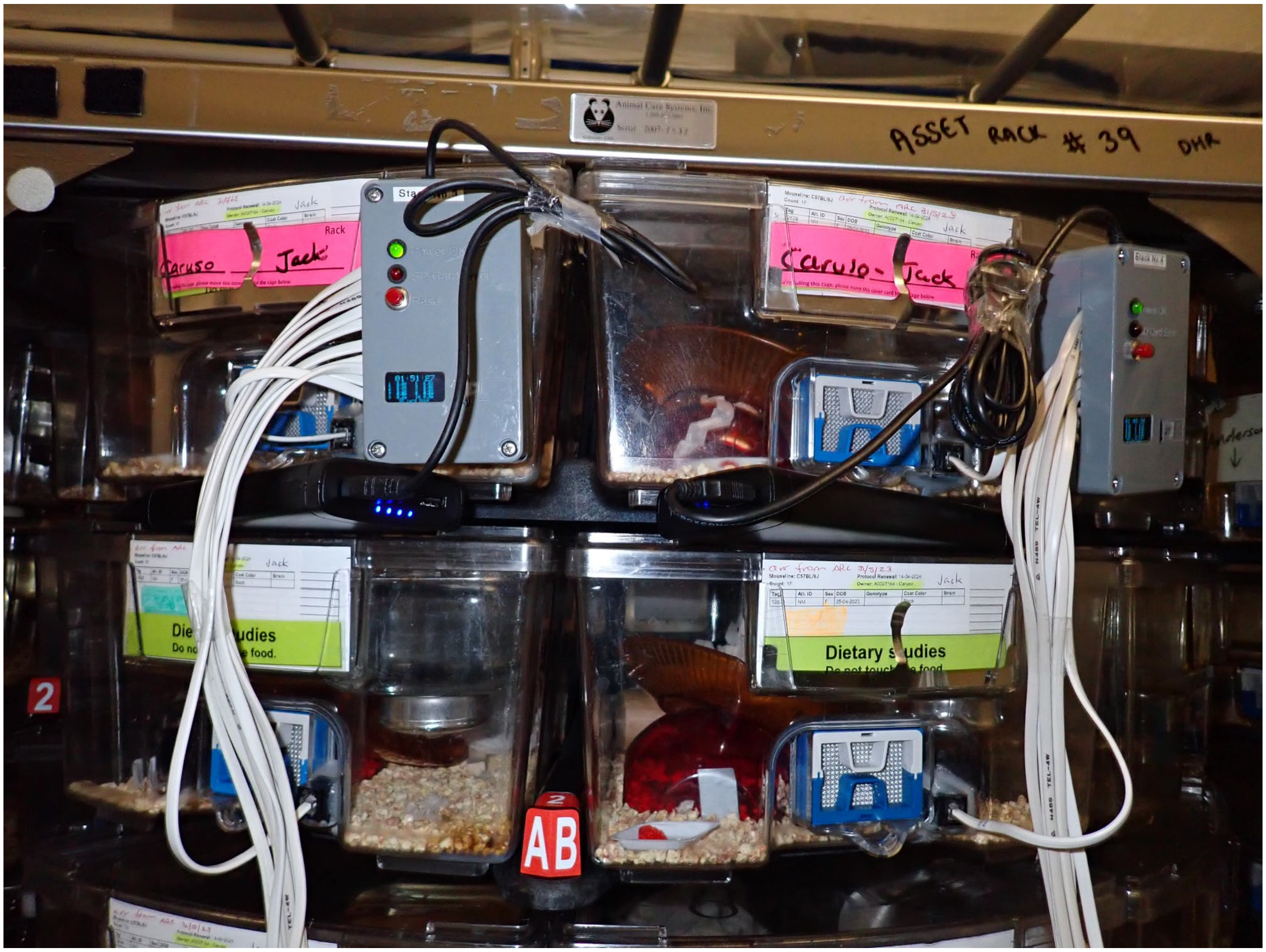

Figure S2. Running Distance analyser

Table S1. Absolute mass percentages of *Undaria pinnatifida* fucoidan (UPF) extract

Fucoidan extract	Neutral (%)	Carbohydrates	Sulfate (%)	Fucoidan (%)	Polyphenols (%)
UPF2022532	46.1		28.3	89.3	<2

Table S2. Carbohydrate breakdown (mass %) of neutral carbohydrates in *Undaria pinnatifida* fucoidan (UPF) extract

Fucoidan extract	Fucose (%)	Xylose (%)	Galactose (%)	Arabinose (%)	Rhamnose (%)
UPF2022532	22.5	0.3	19	0.6	0.6

Table S3. Sequence of primers used for RT-qPCR assay in mouse muscle samples

Gene	Forward primer (5′ to 3′)	Reverse primer (3′ to 5′)
COX2	ATCCCAGGCCGACTAAATCAAG	AGAGCATTGGCCATAGAATAAC
COX4	CTATGTGTATGGCCCCATCC	CAGCGGGCTCTCACTTCTTC
MYH1	GAATGGCAAGACGGTGACTGTG	GGAAGCGTAGCGCTCCTTGAG
MYH2A	ATCAACCAGCAGCTGGACACCA	TCCAGCACGAACATGTGGTGGT
PGC-1α	CGCAGGTCGAATGAAACTGACTT	GTTACCTGCGCAAGCTTCTCTGA
PPAR- γ	CACAATGCCATCAGGTTTGG	GCTGGTCGATATCACTGGAGATC
IGF1	CTGGACCAGAGACCCTTTGC	GGACGGGGACTTCTGAGTCTT
Housekeeping		
β-actin	CCTAAGGCCAACCGTGAAAA	AGCCATACAGGGACAGCACA
GAPDH	AAGGTCGGTGTGAACGGATTTG	TGTAGACCATGTAGTTGAGGTCA

Table S4. Sequence of primers used for bacterial profiling by quantitative PCR

Bacterial group	Forward primer (5′ to 3′)	Reverse primer (3′ to 5′)
Firmicutes phylum	GGAGYATGTGGTTTAATTCGAA-GCA	AGCTGACGACAACCATGCAC
Bacteroidetes phylum	GGAR-CATGTGGTTTAATTCGATGAT	AGCTGACGACAACCATGCAG
Enterobacte-riales order	ATGGCTGTCGTCAGCTCGT	CCTACTTCTTTTGCAACCCACTC
Bifidobacterium spp.	TCGCGTCYGGTGTGAAAG	RCCACATCCAGCRTCCAC
Lactobacillus group	GAGGCAGCAGTAGGGAATCTTC	GGCCAGTTACTACCTC-TATCCTTCTTC
Bacteroides /Prevotella	TCCTACGGGAGGCAGCAGT	CAATCGGAGTTCTTCGTG
Akkermansia muciniphila	CAGCACGTGAAGGTGGGGAC	CCTTGCGGTTGGCTTCAGAT
Clostridium coccoides	ACTCCTACGGGAGGCAGC	GCTTCTTAGTCARGTACCG
Housekeeping		
Total Bacteria	ACTCCTACGGGAGGCAGCAG	ATTACCGCGGCTGCTGG

Forward and reverse sequences for qPCR amplification in mouse faecal samples.
Nucleotide symbols: R = A or G, and Y = C or T.

Table S5. Effects of UPF, diet and exercise on energy intake and anthropometric parameters

Group	**CHOW**	**CHOW+ UPF**	**HFD**	**HFD+ UPF**
Body weight (g)	22.92 ± 0.58	23.06 ± 0.62	27.31 ± 1.06 #	27.87 ± 0.93 #
Energy Intake (Kcal/kg/24h)	14.87 ± 0.41	13.95 ± 0.47	15.98 ± 0.84	15.80 ± 0.77
RP fat (mg)	93.63 ± 9.32	105.60 ± 10.02	469.88 ± 52.58 #	518.13 ± 54.97 #
Gonadal fat (mg)	307.20 ± 95.12	301.73 ± 31.94	1281.75 ± 150.95 #	1351.38 ± 106.47 #
BAT (mg)	54.81 ± 2.32	54.27 ± 2.74	81.94 ± 6.12 #	76.63 ± 6.37 #
Visceral fat (mg)	401.87 ± 25.88	407.33 ± 38.65	1751.63 ± 197.73 #	1869.50 ± 147.79 #
Leg muscle (mg)	232.53 ± 8.12	264.53 ± 7.12 *	267.50 ± 10.50 #	281.13 ± 12.26 #
RP fat %BW (%)	0.41 ± 0.04	0.46 ± 0.04	1.69 ± 0.15 #	1.82 ± 0.18 #
Gonadal fat %BW (%)	1.33 ± 0.07	1.28 ± 0.11	4.55 ± 0.38 #	4.78 ± 0.30 #
BAT %BW (%)	0.24 ± 0.01	0.23 ± 0.01	0.30 ± 0.01 #	0.27 ± 0.01 #
Visceral fat %BW (%)	1.74 ± 0.09	1.74 ± 0.14	6.23 ± 0.50 #	6.60 ± 0.41 #
Skeletal muscle %BW (%)	1.02 ± 0.03	1.14 ± 0.02*	0.98 ± 0.03 #	1.01 ± 0.03 #
Plasma leptin (ng/mL)	1.02 ± 0.19	0.69 ± 0.14	5.08 ± 1.05 #	4.54 ± 0.81 #
Plasma ghrelin (pg/mL)	2.71 ± 0.11	2.68 ± 0.15	2.60 ± 0.14	2.67 ± 0.10
Blood glucose (mmol/L)	9.51 ± 0.19	9.81 ± 0.25	10.48 ± 0.39	10.33 ± 0.36

n = 9-16. Results are expressed as mean ± SE. Data were analysed by one-way ANOVA, followed by post hoc LSD tests.
#, $P<0.05$, overall difference between (CHOW and CHOW+UPF) vs (HFD and HFD+UPF).
*, $P<0.05$, difference between CHOW vs CHOW+UPF and HFD vs HFD+UPF.
BW: body weight; Rp retroperitoneal; BAT: brown adipose tissue; WAT: white adipose tissue.
