# Supplementary figures and images for: Fucoidan from Undaria pinnatifida Enhances Exercise Performance and Increases the Abundance of Beneficial Gut Bacteria in Mice
Source: Mar Drugs. 2024 Oct 29;22(11):485. doi: 10.3390/md22110485 (PMC11595500; doi:10.3390/md22110485)

## Slide 1
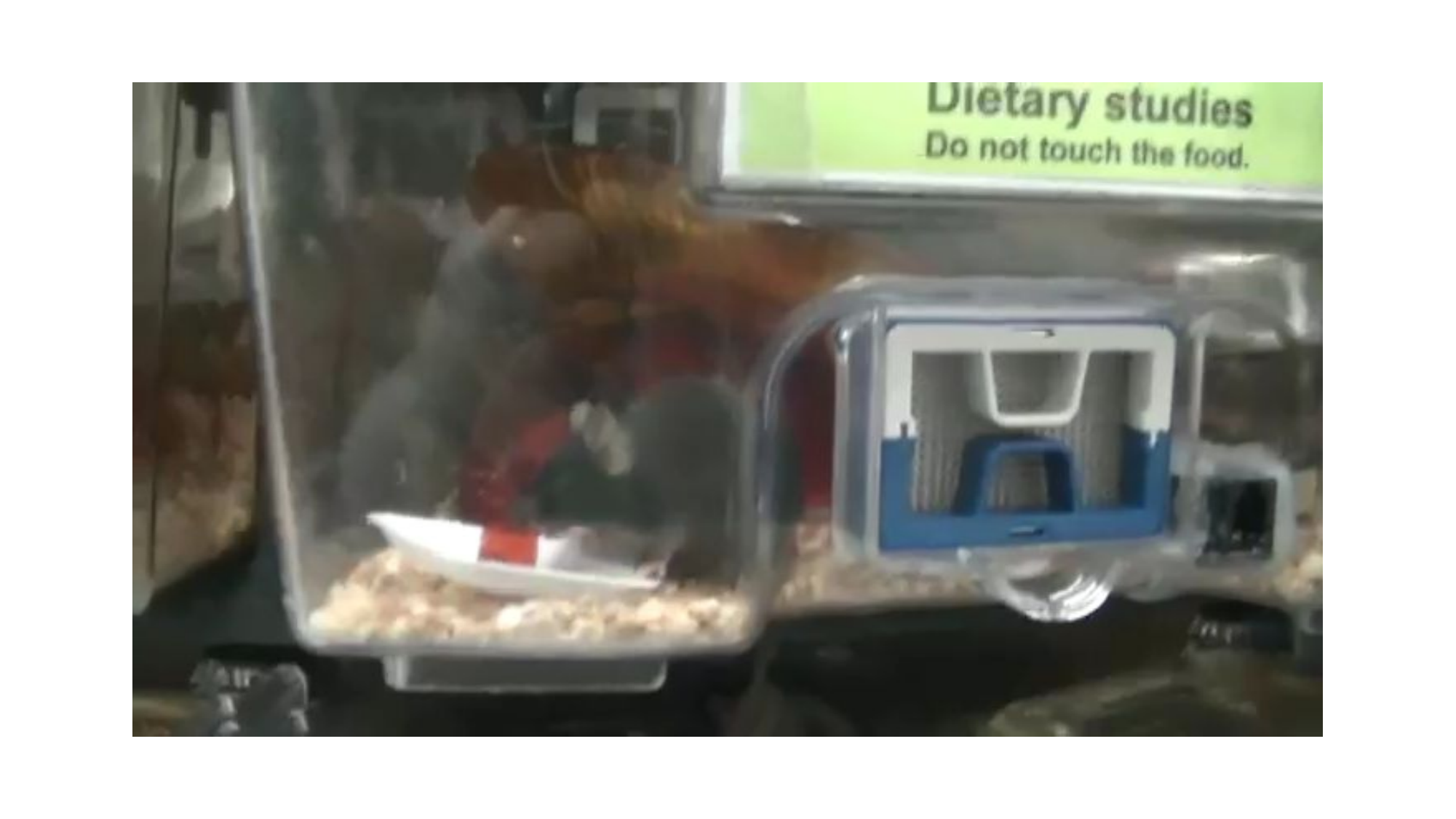

## Slide 2
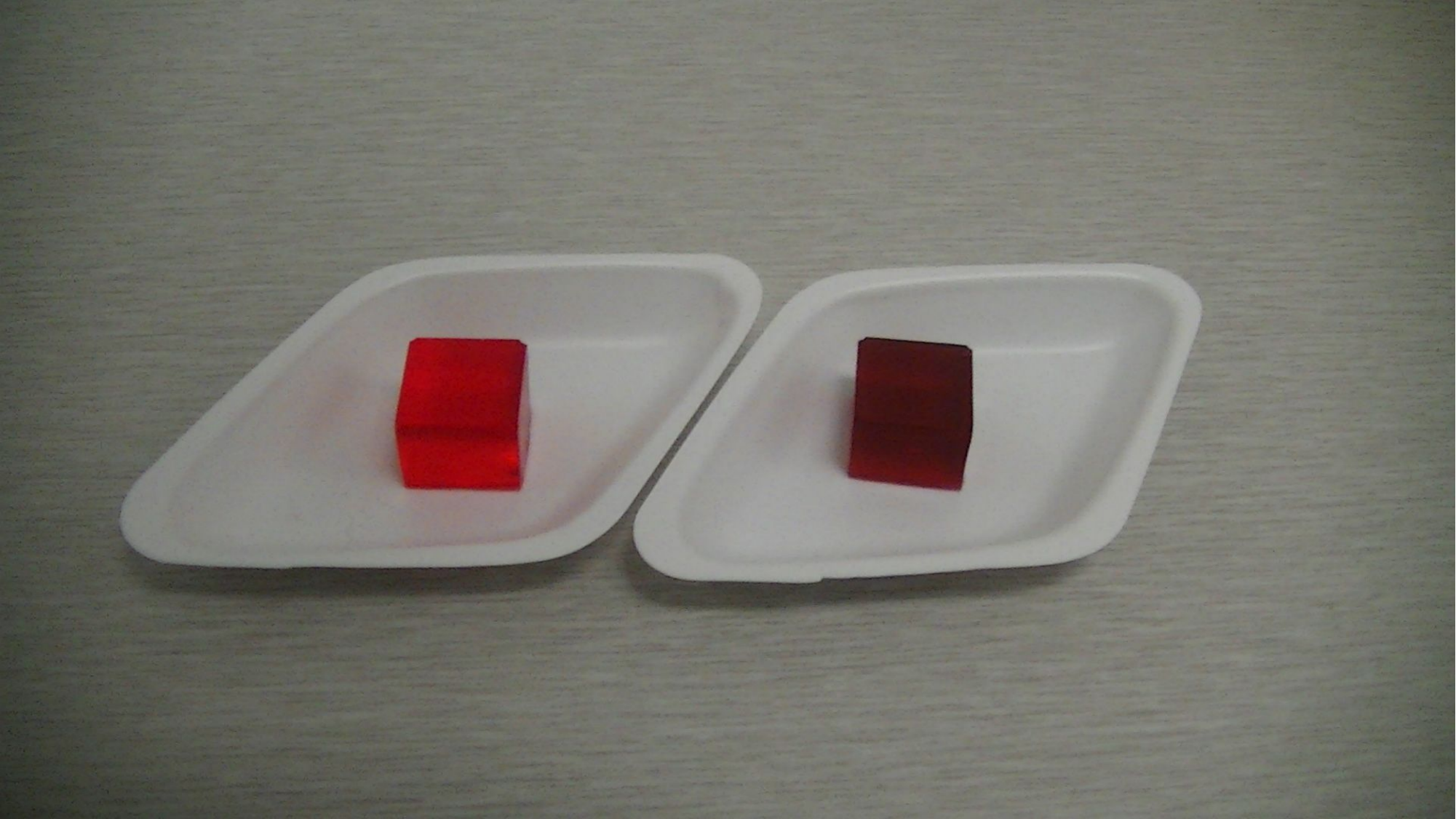

Supplement: Supplementary file 1 [file marinedrugs-22-00485-s001.zip › marinedrugs-3238273-supplementary.pptx]
